# Development of a Consensus-Based Definition of Focused Assessment With Sonography for Trauma in Children

**DOI:** 10.1001/jamanetworkopen.2022.2922

**Published:** 2022-03-18

**Authors:** Aaron E. Kornblith, Newton Addo, Monica Plasencia, Ashkon Shaahinfar, Margaret Lin-Martore, Naina Sabbineni, Delia Gold, Lily Bellman, Ron Berant, Kelly R. Bergmann, Timothy E. Brenkert, Aaron Chen, Erika Constantine, J. Kate Deanehan, Almaz Dessie, Marsha Elkhunovich, Jason Fischer, Cynthia A. Gravel, Sig Kharasch, Charisse W. Kwan, Samuel H. F. Lam, Jeffrey T. Neal, Kathyrn H. Pade, Rachel Rempell, Allan E. Shefrin, Adam Sivitz, Peter J. Snelling, Mark O. Tessaro, William White

**Affiliations:** 1 Department of Pediatrics, University of California, San Francisco, San Francisco; 2 Department of Emergency Medicine, University of California, San Francisco, San Francisco; 3 Department of Medicine, University of California, San Francisco, San Francisco; 4 Department of Bioengineering and Therapeutic Sciences, University of California, San Francisco, San Francisco; 5 Department of Bioengineering, University of California, Berkeley, Berkeley; 6 Department of Pediatrics, Division of Emergency Medicine, Nationwide Children’s Hospital, Columbus, Ohio; 7 Division of Pediatric Emergency Medicine, Department of Pediatric Emergency Medicine, Harbor-UCLA (University of California, Los Angeles) Medical Center, California Pacific Medical Center, Los Angeles; 8 Department of Emergency Medicine, Schneider Children’s Medical Center of Israel, Petah Tikva, Israel; 9 Department of Pediatric Emergency Medicine, Children’s Minnesota, Minneapolis; 10 Division of Pediatric Emergency Medicine, Cincinnati Children’s Hospital Medical Center, Cincinnati, Ohio; 11 Division of Emergency Medicine, Children’s Hospital of Philadelphia, Philadelphia, Pennsylvania; 12 Division of Pediatric Emergency Medicine, Hasbro Children’s Hospital, Rhode Island Hospital, Providence; 13 Division of Pediatric Emergency Medicine, Johns Hopkins Children’s Center, Baltimore, Maryland; 14 Department of Emergency Medicine, Columbia University Vagelos College of Physicians and Surgeons, New York, New York; 15 Division of Emergency and Transport Medicine, Children’s Hospital Los Angeles, Los Angeles, California; 16 Division of Emergency Medicine, The Hospital for Sick Children, University of Toronto, Toronto, Ontario, Canada; 17 Division of Emergency Medicine, Boston Children’s Hospital, Boston, Massachusetts; 18 Department of Pediatrics, Massachusetts General Hospital, Boston; 19 Department of Emergency Medicine, Massachusetts General Hospital, Boston; 20 Department of Pediatric Emergency Medicine, London Health Sciences Centre Children's Hospital, Western University, London, Ontario, Canada; 21 Department of Emergency Medicine, Sutter Medical Center Sacramento, Sacramento, California; 22 Department of Emergency Medicine, Rady Children’s Hospital, University of California, San Diego, San Diego; 23 Department of Pediatrics, Children's Hospital of Eastern Ontario, University of Ottawa, Ottawa, Ontario, Canada; 24 Department of Pediatric Emergency Medicine, Children’s Hospital of New Jersey, Newark Beth Israel Medical Center, Newark; 25 Department of Pediatric Emergency Medicine, Gold Coast University Hospital, Griffith University, Brisbane, Queensland, Australia

## Abstract

**Importance:**

The wide variation in the accuracy and reliability of the Focused Assessment With Sonography for Trauma (FAST) and the extended FAST (E-FAST) for children after blunt abdominal trauma reflects user expertise. FAST and E-FAST that are performed by experts tend to be more complete, better quality, and more often clinically valuable.

**Objective:**

To develop definitions of a complete, high-quality, and accurate interpretation for the FAST and E-FAST in children with injury using an expert, consensus–based modified Delphi technique.

**Design, Setting, and Participants:**

This consensus-based qualitative study was conducted between May 1 to June 30, 2021. It used a scoping review and iterative Delphi technique and involved 2 rounds of online surveys and a live webinar to achieve consensus among a 26-member panel. This panel consisted of international experts in pediatric emergency point-of-care ultrasonography.

**Main Outcomes and Measures:**

Definitions of complete, high-quality, and accurate FAST and E-FAST studies for children after injury.

**Results:**

Of the 29 invited pediatric FAST experts, 26 (15 men [58%]) agreed to participate in the panel. All 26 panelists completed the 2 rounds of surveys, and 24 (92%) participated in the live and asynchronous online discussions. Consensus was reached on FAST and E-FAST study definitions, and the panelists rated these 5 anatomic views as important and appropriate for a complete FAST: right upper-quadrant abdominal view, left upper-quadrant abdominal view, suprapubic views (transverse and sagittal), and subxiphoid cardiac view. For E-FAST, the same FAST anatomic views with the addition of the lung or pneumothorax view were deemed appropriate and important. In addition, the panelists rated a total of 32 landmarks as important for assessing completeness. Similarly, the panelists rated 14 statements on quality and 20 statements on accurate interpretation as appropriate.

**Conclusions and Relevance:**

This qualitative study generated definitions for complete FAST and E-FAST studies with high image quality and accurate interpretation in children with injury. These definitions are similar to those in adults with injury and may be used for future education, quality assurance, and research. Future research may focus on interpretation of trace volumes of abdominal free fluid and the use of serial FAST.

## Key Points

**Question:**

How do experts define a complete, high-quality, and accurate interpretation of Focused Assessment With Sonography for Trauma (FAST) for children with injury?

**Findings:**

In this qualitative study involving 26 international, pediatric emergency point-of-care ultrasonography experts, a consensus was achieved on the definitions of FAST and extended FAST studies using a modified Delphi technique. The definitions included ultrasonographic views, landmarks, and patient-specific factors that affect interpretation accuracy.

**Meaning:**

The definitions established in this study may assist clinicians in completing the necessary components of FAST or extended FAST for children with injury and may be used for future education, quality assurance, and research.

## Teaser

This qualitative study describes the use of a modified Delphi process for soliciting the input of a panel of experts in defining a standardized examination protocol for pediatric patients with a blunt torso trauma.

## Introduction

Intra-abdominal injury after blunt abdominal trauma is a leading cause of preventable deaths in children in the US.^1^ However, early and accurate diagnosis of intra-abdominal injury in children is challenging. Current diagnostic strategies are suboptimal because of the trade-off between missed injury and resource overutilization, including children’s exposure to ionizing radiation from computed tomography (CT) scans.

Focused Assessment With Sonography for Trauma (FAST) is a point-of-care ultrasonography (POCUS) study that uses no radiation. The FAST method was introduced in the US in the 1990s to describe a set of ultrasonographic views for the rapid evaluation of free fluid (hemorrhage) in patients with injury.^2^ In adult patients, use of FAST decreases time to surgical intervention, patient length of stay, surgical complications, and CT scan and diagnostic peritoneal lavage rates.^3^ However, compared with CT, the test characteristics of FAST have variable reliability and accuracy for identifying intra-abdominal injury in children.^4,5,6,7^ For this reason, FAST has not been ubiquitously incorporated into diagnostic strategies for children with injury.^6,8^

Previous studies have suggested that clinicians with more experience in performing FAST in children have higher diagnostic yields.^9,10^ In addition, experienced clinicians have been found to be more likely to capture complete, high-quality images and feel confident about integrating their results into their clinical strategy.^11,12^ Currently, there is no agreed-on standard for a complete protocol, adequate image quality, and accurate interpretation for FAST in children with injury.^13^ This lack of a standardized pediatric FAST definition is a critical factor in the variability in its use, image quality, and diagnostic accuracy. Therefore, we conducted this qualitative study to define a complete, high-quality, and accurate interpretation for FAST and extended FAST (E-FAST) in children with injury using an expert, consensus–based modified Delphi technique.

## Methods

For this qualitative study, the 2-round, mixed-methods, modified consensus Delphi technique^14^ was conducted between May 1 to June 30, 2021, and consisted of 2 web-based surveys per guidelines^15^ and 1 live webinar consensus meeting between rounds. The institutional review board at the University of California, San Francisco approved this study. Panelists provided verbal consent to participate and were allowed to withdraw at any time. The reporting of this study follows the Consolidated Criteria for Reporting Qualitative Research (COREQ) reporting guideline.^16^

The modified Delphi technique is a consensus-based approach that systematically assembles statements from a group of experts.^15^ The method is iterative and encourages participants to share opinions but uses anonymity to reduce participants’ dominant impact. The RAND/UCLA Appropriateness Method is a modified Delphi technique that, unlike the original Delphi, allows panelists the opportunity to discuss their judgments between rounds.^17^

### Panel Selection and Survey Instrument

Those of us in the Steering and Executive Writing Committee (A.E.K., N.A., M.P., A. Shaahinfar, M.L.-M., N.S., and D.G.) outlined the objectives of the FAST consensus panel and invited an international group of pediatric emergency POCUS experts from the P2Network Research Committee writing group.^18^ The P2Network is a platform for sharing information and collaborating on pediatric emergency medicine POCUS initiatives. The 26-member panel was chosen to represent a geographically diverse sampling of experts. Age and race and ethnicity data were not collected. Experts were defined as those who completed 1500 or more POCUS studies or served in an institutional POCUS leadership role.^19,20^ The intended participants received an email outlining the objectives, methods, and requirements of the study.

**Figure 1.**
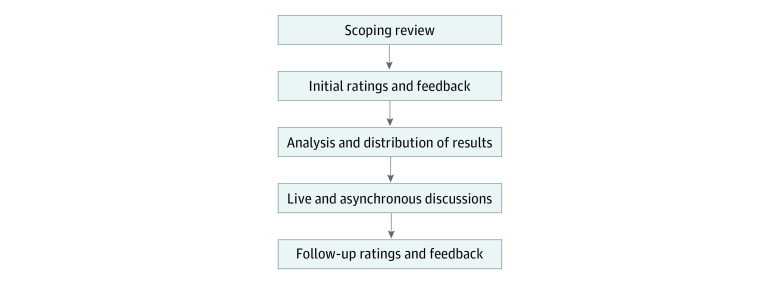
Study Design With a Modified Delphi Approach

The Steering and Executive Writing committee conducted an initial scoping review to assess contemporary and historical literature that could be used to define a complete FAST protocol, study views, landmarks, adequate image quality, and accurate study interpretation (Figure 1). A parallel search strategy from a previous systematic review of pediatric FAST was used.^6^ The committee searched PubMed from January 1, 1968, to December 31, 2020, using the following Medical Subject Headings terms: hemorrhage, bleeding, sensitivity, specificity, ultrasound, ultrasonography, focused assessment with sonography for trauma, and protocol. The committee included studies that specified pediatric patients and English language titles. All panel participants were allowed to suggest reports and other published content during the first consensus round. In addition, the committee reviewed specialty-specific society guidelines, consensus statements for FAST in adult patients with injury, POCUS curricula, and frequently cited textbooks and websites.^21,22,23^

Based on the scoping review, the initial series of survey items was developed and then organized into the following domains: study definition, completeness (views and landmarks), image quality, and interpretation accuracy. The online survey instrument (REDCap; Vanderbilt University) was initially pilot tested by an emergency medicine POCUS expert who did not participate in the study.

### First-Round Survey

Panelists received email instructions, the scoping review literature summary, and an anonymous survey link. Panelists were given 10 days to rank the appropriateness of FAST and E-FAST definitions, landmarks, quality, and accuracy statements on a scale of 1 to 9, with 1 indicating extremely inappropriate and 9 indicating extremely appropriate. All survey items included space for comments, reflection on the phrasing of an item, or a description of the relevant studies that played a role in the panelist’s response.

### Live Webinar Discussion

After the initial survey, the panelists were provided with a summary of responses that described the means, medians, IQRs, and accompanying histograms for appropriateness ratings. The free-text responses were also summarized, and each participant received their individual responses. To prevent bias, the committee maintained the anonymity of replies from other participants.

During the live webinar discussion on May 17, 2021, panelists discussed the first-round results and anonymously provided verbal or written feedback on survey items. In addition, all panelists were given 7 days to access an online document (Google Docs; Google) to anonymously and asynchronously comment on all results, respond to other panelists, suggest new items, and edit existing survey items. The committee iteratively reviewed, refined, discussed, and summarized into statements the input from the webinar discussion and online document.

### Second-Round Survey

The committee adjusted the initial survey items for content and phrasing according to participant feedback. No survey items were removed. Panelists were sent a hyperlink to the second-round survey and asked to rate the FAST and E-FAST definitions, landmarks, quality, and accuracy statements for appropriateness (using a 1 to 9 scale, with 1 indicating extremely inappropriate and 9 indicating extremely appropriate); completeness according to evaluations, views, and landmarks; and importance (using a 1 to 9 scale, with 1 indicating not all important and 9 indicating extremely important).

The panelists had 10 days to complete the second survey. The committee then reviewed all of the survey results, final statements, and comments. Final statements within similar domains were merged into hybrid statements and reviewed for accuracy by all panelists.

### Statistical Analysis

The committee used the RAND/UCLA Appropriateness Method to analyze the survey responses from the first round and the comments from the webinar and online document in the second round.^17^ In round 1, survey responses were grouped according to numeric ratings and recurring themes. Then, the round 2 comments were iteratively reviewed, refined, discussed, and summarized into statements for final rankings.

Panel consensus was ascertained after round 2 using the interpercentile range adjusted for symmetry (IPRAS) method, as described in the RAND/UCLA Appropriateness Method User’s Manual.^17^ All quantitative responses were evaluated for agreement using the IPRAS method and presented with their median ratings and IQRs. Median ratings were classified as appropriate or important (7-9), uncertain (3.5-6.5), and inappropriate or not important (1-3) for each survey item in which there was no disagreement (eTable 1 in the Supplement). The IPRAS method used the distribution of results instead of a threshold to establish agreement. Disagreement on survey items was defined where the distribution of responses was bimodal or where the calculated interpercentile ranges (difference between 30th and 70th percentiles) were higher than the IPRAS.^17,24^ Moreover, the survey results reported the proportion of respondents who considered items as appropriate or important.

## Results

Of the 29 invited pediatric FAST experts, 26 (90%) agreed to participate in the panel and 3 responded as unavailable. All 26 panelists (11 women [42%] and 15 men [58%]) completed the surveys in both rounds, and 24 of 26 panelists (92%) participated in the live and asynchronous online discussions. The FAST consensus panel consisted of physicians with board certification in 4 specialties: emergency medicine, pediatrics, pediatric emergency medicine, and internal medicine (Table 1). More than half of the panelists had greater than 5 years of postgraduate POCUS experience, and most panelists worked at a Level 1 trauma center.

**Table 1.** Characteristics of the Consensus Panel

Characteristic	Participants, No. (%) (N = 26)
Board certifications	
Emergency medicine	6 (23)
Pediatrics	20 (77)
Pediatric emergency medicine	24 (92)
Internal medicine	1 (4)
No. of postgraduate years of POCUS experience^a^	
0-2	2 (8)
3-5	8 (31)
6-9	9 (35)
10-14	7 (27)
No. of POCUS examinations performed or reviewed per wk	
1-5	4 (15)
6-10	1 (4)
>10	21 (81)
No. of years teaching or clinically instructing POCUS	
3-5	8 (31)
6-9	12 (46)
10-14	6 (23)
Practice at a level 1 trauma center	22 (85)
Completed POCUS fellowship	22 (85)
Country of practice	
United States	20 (77)
Canada	4 (15)
Australia	1 (4)
Israel	1 (4)

Abbreviation: POCUS, point-of-care ultrasonography.

^a^ Not including fellowship or residencies; 23 of 26 physicians (88%) completed more than 1500 examinations in their career. All 26 physicians have either fulfilled this requirement or held an ultrasonography-related leadership role in their department (eg, ultrasonography director, POCUS director, and POCUS fellowship director).

A summary of the panel responses and changes from the first round to the second round of surveys is presented in eTable 2 in the Supplement. Briefly, round 1 included 125 survey items, of which 84 were deemed appropriate and reached consensus. In addition to suggested modifications, 26 new survey items were included in the revised survey for a total of 151 items. An agreement was achieved on FAST and E-FAST study definitions, protocol evaluations, views, and landmarks; 14 statements on image quality; and 20 statements on accurate interpretation.

### Proposed Study Definitions and Evaluations

The consensus definitions for FAST and E-FAST (Box) were rated as appropriate, with both definitions receiving a median (IQR) rating of 9 (8-9). There were several notable comments regarding the FAST and E-FAST definitions. Some panelists commented that the word “limited” might suggest a lack of quality; therefore, replacing the word with “focused” was proposed. Ultimately, the panel decided to keep “limited” to avoid repeating “focused,” which was used to define the acronym FAST. Similarly, the term “peritoneal” was suggested instead of “intraperitoneal.” However, the FAST or E-FAST does not evaluate the retroperitoneal cavity, and thus “intraperitoneal” was deemed as the more precise choice.

Box. **Definitions and Hybrid Summary Statements for FAST and E-FAST for Children With Injury**

**Definitions**

FAST is a noninvasive, limited, POCUS study used in patients after abdominal or chest trauma to detect intraperitoneal, pericardial, or pleural free fluid.
E-FAST is a noninvasive, limited, POCUS study used in patients after abdominal or chest trauma to detect intraperitoneal, pericardial, or pleural free fluid and includes a thoracic examination for pneumothorax.

**Completeness**

1. A complete negative study result must include an adequate evaluation of all anatomic views. In contrast, a positive study result must consist of a thorough evaluation of each anatomic region, with at least 1 view demonstrating abdominal, thoracic, or pericardial free fluid (pathology).
  - FAST: right upper-quadrant abdominal view, left upper-quadrant abdominal view, transverse and sagittal suprapubic view, and pericardial view.
  - E-FAST: FAST and lung or thoracic view.
2. There are specific views and anatomic landmarks that are necessary to ensure an accurate interpretation. Some anatomic landmarks are not as important as others to provide an adequate study and accurate interpretation. Key anatomic landmarks are required to mark a study as complete. Diagnostic performance suffers when specific anatomic landmarks are missed, and an incomplete study could preclude an assessment for pathology.

**Quality**

1. Appropriate transducer selection is dependent on the view, patient age, and body habitus.
2. Adequate transducer manipulation, including fanning or interrogation, is required to completely visualize landmarks within a given view and is one of the factors associated with view quality.
3. Poor gain, inappropriate depth, or sonographic artifacts in an image can obscure landmarks or free fluid visualization.
  - Improperly gained images may be too dark or too bright to assess for free fluid. When evaluating for free fluid, the relative echogenicity of intravascular blood or urine in the bladder can optimize gain.
  - Excessive or insufficient depth can significantly limit study quality and user interpretation. Depth should be optimized for viewing the anatomic landmarks within the focal zone.
  - Use of sufficient gel and adequate skin contact are factors associated with image quality.

**Interpretation Accuracy**

1. Positive FAST or E-FAST result: a study can be considered positive for free fluid when a single view or landmark within a view indicates the presence of intraperitoneal, pericardial, or intra-thoracic free fluid. A FAST study may be considered positive for free fluid even if the operator has not visualized one or more landmarks in each anatomic region.
  - Free fluid will appear on ultrasonography as an anechoic region within the intraperitoneal, intrathoracic, or pericardial spaces. Fluid appearance may become hyperechoic or heterogeneous with coagulation.
  - Trace free fluid in the pelvis may be considered a positive study.
  - The cardiac view is used to identify the presence of pericardial effusion and cardiac activity. The subxiphoid or parasternal views are adequate for interpretation of the pericardial window. Multiple cardiac views may increase the likelihood of identifying pericardial effusion.
  - Pneumothorax evaluation should rule out clinically significant pneumothoraces. The position of free air accumulation and accurate interpretation of pneumothorax are affected by patient positioning.
2. Negative FAST or E-FAST result: a study can be considered negative for free fluid if the study has adequate completeness and quality and if no free fluid can be seen at any landmarks.
3. Limitations: a limitation of FAST is that a small volume of free fluid may be difficult to visualize, especially in young children or those with larger body habitus.
  - False-negative results may occur when the study is performed too early in the clinical course for free fluid detection. If the gain is not adjusted for posterior acoustic enhancement, clinicians may report false-negatives on the suprapubic view. Serial studies may improve the diagnostic yield in patients with active hemorrhage.
  - False-positive results on the suprapubic view could include trace amounts of free fluid, intraluminal fluid (inside bowel), seminal vesicles, iliac vessels, psoas muscles, or shadow artifact.
4. Patient factors for accuracy: age and sex alter the importance of landmark completeness in the pelvic view.
  - In prepubertal children, a good-quality FAST requires a complete suprapubic view.
  - In postpubertal children, a good-quality FAST requires a complete right upper-quadrant view. Similarly, a complete suprapubic view includes sufficient interrogation of spaces that are posterior to the bladder and uterus in postpubertal female children.

Abbreviations: E-FAST, Extended Focused Assessment With Sonography for Trauma; FAST, Focused Assessment With Sonography for Trauma; POCUS, point-of-care ultrasonography.

Intraperitoneal free fluid, pericardial fluid, and pleural fluid were considered essential evaluations for FAST. These same 3 evaluations, with the addition of pneumothoraces, were deemed essential for E-FAST. Inclusion of the evaluation for cardiac activity or cardiac standstill was unclear after 2 rounds of surveys for FAST (median [IQR] rating, 6 [4.25-7.75]) and E-FAST (median [IQR] rating, 6 [4.25-9]). Evaluations for pneumopericardium and bladder injury were not considered important for FAST or E-FAST.

### Completeness by Views

**Figure 2.**
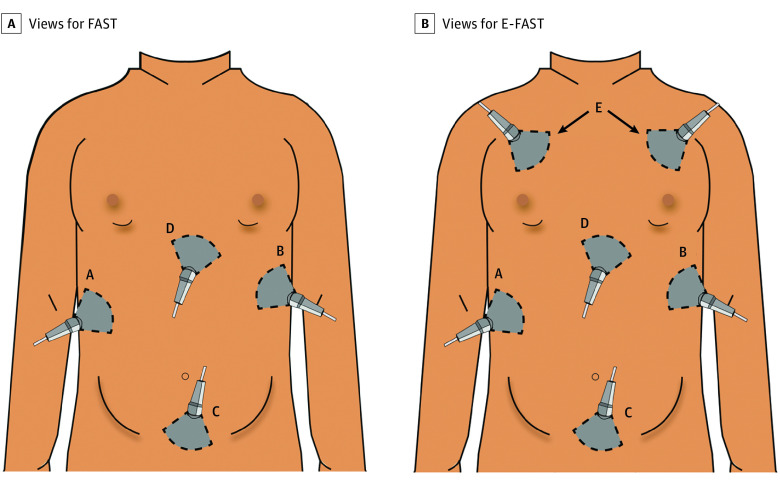
Views Appropriate and Important for Focused Assessment With Sonography for Trauma (FAST) and Extended FAST (E-FAST) Views for FAST include right upper-quadrant abdominal (A); left upper-quadrant abdominal (B); suprapubic, both transverse and sagittal (C); and subxiphoid cardiac (D) views. Views for E-FAST include the FAST plus the lung or pneumothorax (E) views.

The panelists found that a complete FAST is dependent on whether it has a negative or positive result (Box). A negative FAST result must include an adequate evaluation of all FAST views. In contrast, a positive FAST result must evaluate each anatomic region, with at least 1 view demonstrating abdominal, thoracic, or pericardial free fluid (pathology). To examine the anatomic regions, the panelists found the following views to be appropriate and important for FAST: right upper-quadrant abdominal view, left upper-quadrant abdominal view, suprapubic views (both transverse and sagittal views), and subxiphoid cardiac view (Figure 2A). The same views were appropriate and important for E-FAST with the addition of the lung or pneumothorax view (Figure 2B). The parasternal cardiac long view was rated as appropriate for both FAST and E-FAST, but its importance did not reach an agreement. The panel commented that the parasternal long cardiac view could be considered an acceptable substitute if the subxiphoid cardiac view was technically limited or challenging to perform.

Both suprapubic views (transverse and sagittal) were found to be appropriate and important. In contrast to the cardiac views, 19 panelists (73%) responded that 2 suprapubic views (transverse and sagittal views) were required, whereas 7 panelists (27%) responded that 1 suprapubic view was sufficient.

### Completeness by Landmarks

The panelists rated 32 anatomic landmarks for the FAST and E-FAST ultrasonographic views. The panelists agreed that essential anatomic landmarks within each view were necessary to ensure a complete and accurate interpretation of FAST. However, they did not agree that some anatomic landmarks were not as important as others for a complete FAST. For the right upper-quadrant abdominal view, the panelists agreed on the importance of the Morison pouch (median [IQR] rating, 9 [9-9]), caudal edge or tip of the liver (median [IQR] rating, 9 [9-9]), superior pole of the kidney (median [IQR] rating, 9 [9-9]), inferior pole of the kidney (median [IQR] rating, 9 [9-9]), subdiaphragmatic space (median [IQR] rating, 9 [9-9]), and supradiaphragmatic space (median [IQR] rating, 9 [8.25-9]) (Table 2).^25,26^ In contrast, the panelists were uncertain about the importance of viewing the Glisson (liver) capsule (median [IQR] rating, 5 [2-8.75]), spine (median [IQR] rating, 6 [5.25-7.75]), psoas (median [IQR] rating, 3.5 [2-5]), and paracolic gutter (median [IQR] rating, 4.5 [2-6]) within the right upper-quadrant abdominal view (Table 2).

**Table 2.** Consensus Panel Ratings of Final Landmark Items and Ultrasonographic Views in FAST and E-FAST

Landmark^**a**^	Median importance rating (IQR)
Right upper-quadrant abdominal view	
Morison pouch (hepatorenal recess)^b^	9 (9-9)
Caudal edge or tip of the liver^b^	9 (9-9)
Superior pole of the kidney^b^	9 (9-9)
Inferior pole of the kidney^b^	9 (8-9)
Subdiaphragmatic space^b^	9 (9-9)
Supradiaphragmatic space^b^	9 (8.25-9)
Glisson (liver) capsule	5 (2-8.75)
Paracolic gutter	4.5 (2-6)
Spine	6 (5.25-7.75)
Psoas	3.5 (2-5)
Left upper-quadrant abdominal view	
Spleen^b^	9 (9-9)
Tip of the spleen^b^	9 (9-9)
Perisplenic space^b^	9 (9-9)
Splenorenal recess^b^	9 (9-9)
Gastrosplenic recess	4.5 (3-7)
Subdiaphragmatic space^b^	9 (9-9)
Supradiaphragmatic space^b^	9 (8-9)
Superior pole of the kidney^b^	9 (9-9)
Inferior pole of the kidney^b^	9 (7-9)
Paracolic gutter	5 (2.25-5.75)
Spine	5.5 (5-7)
Transverse suprapubic view	
Posterior wall of the bladder^b^	9 (9-9)
Anterior wall of the bladder^b^	9 (8-9)
Bladder-colon interface	7 (5-9)
Psoas	3 (2-5)
Bladder-prostate interface (for male sex)^b^	6.5 (4.25-7.75)
Seminal vesicles (for male sex)	5 (3-6.75)
Ovaries (for female sex)	2 (1.25-3.75)
Uterus (for female sex)	7 (5-8)
Pouch of Douglas or rectouterine space (for female sex)^b^	9 (7.25-9)
Sagittal suprapubic view	
Posterior wall of the bladder^b^	9 (9-9)
Anterior wall of the bladder^b^	9 (8.25-9)
Bladder-colon interface	7 (5-9)
Psoas	2.5 (2-4)
Bladder-prostate interface (for male sex)	5 (3.25-7)
Seminal vesicles (for male sex)	3 (2-5.75)
Ovaries (for female sex)	2.5 (1-3)
Uterus (for female sex)^b^	7 (6-8)
Pouch of Douglas or rectouterine space (for female sex)^b^	9 (8-9)
Pericardial view	
Pericardial border	
Apical^b^	9 (7-9)
Anterior^b^	9 (7.25-9)
Posterior^b^	9 (8-9)
Hepatic pericardial interface^b^	9 (6.25-9)
Left atrium	7 (4-9)^c^
Right atrium	6.5 (3.25-8)
Left ventricle^b^	8.5 (7-9)
Right ventricle^b^	8.5 (6.25-9)
Ascending thoracic aorta	2.5 (1.25-5)
Descending thoracic aorta	5 (2-7)
Lung or thoracic view	
Rib spaces	
At least 1^b^	9 (7-9)
At least 2^b^	8.5 (7-9)
At least 3	8 (3.25-9)^c^
Intercostal muscles	5 (2-7.75)
Pleural sliding	
By B-mode^b^	9 (9-9)
By M-mode	5 (3-6)
Lung point	5 (3-8.75)
Pleural line^b^	9 (9-9)
A line	6 (5-9)
Z line	3.5 (1.25-5.75)
Diaphragm	3.5 (1-7.75)

Abbreviations: E-FAST, Extended Focused Assessment With Sonography for Trauma; FAST, Focused Assessment With Sonography for Trauma.

^a^ Landmarks with a score of 7 to 9 were rated as important; 3.5 to 6.5, uncertain; and 1 to 3, not important for a complete Focused Assessment With Sonography for Trauma examination.

^b^ Landmark ranked as important.

^c^ Disagreement in importance ranking per interpercentile range was adjusted for symmetry.

For the left upper-quadrant abdominal view, the following landmarks were rated important: spleen (median [IQR] rating, 9 [9-9]), tip of the spleen (median [IQR] rating, 9 [9-9]), perisplenic space (median [IQR] rating, 9 [9-9]), splenorenal recess (median [IQR] rating, 9 [9-9]), subdiaphragmatic space (median [IQR] rating, 9 [9-9]), supradiaphragmatic space (median [IQR] rating, 9 [8-9]), superior pole of the kidney (median [IQR] rating, 9 [9-9]), and inferior pole of the kidney (median [IQR] rating, 9 [7-9]).^27^ However, the panelists were uncertain about the importance of the gastrosplenic recess (median [IQR] rating, 4.5 [3-7]), paracolic gutter (median [IQR] rating, 5 [2.25-5.75]), and spine (median [IQR] rating, 5.5 [5-7]) in the left upper-quadrant abdominal view (Table 2).

The suprapubic view was divided into the transverse and sagittal suprapubic anatomic views. For the transverse and sagittal suprapubic view, the panelists agreed on the importance of viewing the anterior and posterior bladder walls and the pouch of Douglas (rectouterine space). In contrast, the panel was uncertain about the importance of the psoas (median [IQR] rating, 3 [2-5]), bladder-prostate interface (median [IQR] rating, 6.5 [4.25-7.75]), and seminal vesicles (median [IQR] rating, 5 [3-6.75]) for the transverse view.^28,29^ The psoas (median [IQR] rating, 2.5 [2-4]), seminal vesicles (median [IQR] rating, 3 [2-5.75]), and ovaries (median [IQR] rating, 2.5 [1-3]) for the sagittal view were rated as unimportant. The bladder-colon interface was rated as important on the transverse view (median [IQR] rating, 7 [5-9]) but had uncertain importance on the sagittal view (median [IQR] rating, 7 [5-9]). In contrast, the uterus had uncertain importance on the transverse view (median [IQR] rating, 7 [5-8]) but was rated as important on the sagittal view (median [IQR] rating, 7 [6-8]). The panel also rated the transverse view as more important for postpubescent boys and the sagittal view as more important for postpubescent girls. The panelists commented that 1 view might be sufficient, but 2 views may optimize the visualization of all landmarks.

For the pericardial view, the following landmarks were deemed important: apical pericardial border (median [IQR] rating, 9 [7-9]), anterior pericardial border (median [IQR] rating, 9 [7.25-9]), posterior pericardial border (median [IQR] rating, 9 [8-9]), hepatic pericardial interface (median [IQR] rating, 9 [6.25-9]), left ventricle (median [IQR] rating, 8.5 [7-9]), and right ventricle (median [IQR] rating, 8.5 [6.25-9]).^22,30^ The left atrium (median [IQR] rating, 7 [4-9]), right atrium (median [IQR] rating, 6.5 [3.25-8]), and ascending thoracic aorta (median [IQR] rating, 2.5 [1.25-5]) had unclear importance as landmarks within the pericardial view. The panelists did not reach an agreement on the importance of the descending thoracic aorta (median [IQR] rating, 5 [2-7]), as some experts noted that this landmark was used to differentiate between pericardial and pleural effusion (Table 2). The panel commented that 1 cardiac view might be sufficient if the landmarks were visualized, which was often dependent on the patient’s body habitus.

For the lung or thoracic view of E-FAST, the panel rated rib spaces (at least 1) (median [IQR] rating, 9 [7-9]), rib spaces (at least 2) (median [IQR] rating, 8.5 [7-9]), pleural sliding by B-mode (median [IQR] rating, 9 [9-9]), and pleural line (median [IQR] rating, 9 [9-9]) as important. In contrast, rib spaces (at least 3) (median [IQR] rating, 8 [3.25-9]), intercostal muscles (median [IQR] rating, 5 [2-7.75]), pleural sliding on M-mode (median [IQR] rating, 5 [3-6]), lung point (median [IQR] rating, 5 [3-8.75]), A lines (median [IQR] rating, 6 [5-9]), Z lines (median [IQR] rating, 3.5 [1.25-5.75]), and diaphragm (median [IQR] rating, 3.5 [1-7.75]) had an uncertain importance for the lung view^21,31^(Table 2).

### Image Quality

Consensus was reached on 14 quality statements, with no disagreements in the second survey. These 14 quality statements were converted into 3 hybrid statements (Box). After round 1, the panelists included image quality related to optimizing depth and gain, transducer selection, and ultrasonography system settings. Other than improving the factors associated with the ultrasonography machine, the panel wanted clinicians to optimize image quality through proper probe manipulation and skin contact through gel use. Similarly, the panelists wanted the definition of free fluid to capture complex fluid collections with a heterogeneous hyperechoic or hypoechoic appearance (eg, blood clot).

### Interpretation Accuracy

The panel reached an agreement on 20 interpretation of accuracy statements in the second survey, which were converted into 4 hybrid statements (Box). Two statements on interpretation accuracy did not reach an agreement but created a divide between the 26 panelists.

One statement, “A FAST study can be considered a qualified negative if the operator does not adequately visualize one or more landmarks,” was rated by 11 panelists as appropriate, whereas 12 panelists rated it as inappropriate. Panelists who agreed with the statement wanted to recognize the limitations of FAST and that patient-level factors may not allow a complete visualization of all landmarks. In contrast, panelists who disagreed with the statement suggested that ranked landmarks could lead to suboptimal FAST studies. The other statement, “Trace free fluid in the pelvis may be considered a negative study,” was rated by 9 panelists as appropriate, whereas 10 panelists rated it as inappropriate. Panelists who agreed with the statement emphasized that trace free fluid could be considered physiological in specific pediatric populations. However, those panelists who disagreed with the statement were unclear on how clinicians could differentiate between physiological and pathological free fluid while acknowledging that the FAST result, whether positive or negative, should not uniquely dictate clinical next steps.^32^

## Discussion

The FAST consensus panel of heterogeneous international experts developed comprehensive definitions for a complete, high-quality, and accurate interpretation of FAST and E-FAST for children with injury. The definitions have implications for clinical use and quality improvement review and provide standard definitions for research on injuries in children. The panelists defined FAST and E-FAST as congruent with the current working definitions for adults with injury.^13,33^

The protocol definition of FAST has undergone alterations to match advances in POCUS applications. For example, FAST was initially proposed as Focused Abdominal Sonography for Trauma^34^; however, with the addition of the cardiac view for evaluating pericardial fluid, FAST transformed to Focused Assessment With Sonography for Trauma.^13^ Similarly, advances in thoracic POCUS and the addition of pneumothorax evaluation led to the expansion of E-FAST.^31^ During the panel discussion, several panelists suggested adding novel POCUS applications to the FAST and E-FAST definitions, such as musculoskeletal evaluation for fracture. However, without sufficient evidence for these novel applications in children, the panel agreed to keep the definitions congruent with the protocol definitions for adults with injury.

During the panel discussion, one area of interest was the definition of FAST compared with the definition of E-FAST. Most panelists agreed with the working definition, but there were substantial discussions regarding the evaluation for hemothorax.^35^ Some panelists considered pleural evaluation only in the E-FAST definition. This opinion stemmed from a conceptualization of the 3 distinct body cavities and consideration for procedure reimbursement in E-FAST, including the *Current Procedural Terminology* (*CPT*) coding schema. The *CPT* schema divides ultrasonographic studies into body cavities, including abdominal, cardiac, and thoracic. Therefore, the addition of a pleural evaluation may alter the reimbursement criteria. However, the historical use of the FAST included the evaluation for hemothorax; thus, the panel agreed to include hemothorax evaluation in both the FAST and E-FAST definitions.^13^ Conversely, the panel discussed replacing the E-FAST definition and incorporating pneumothorax into the FAST definition. However, given that most children with injury are treated in general emergency departments,^36^ where many practitioners train using the adult context or definitions of FAST and E-FAST, it was decided that both definitions would remain unchanged to avoid confusion between pediatric and adult POCUS studies. These panel discussions suggest that the POCUS community may consider developing a novel FAST *CPT* code to avoid the constraints of the current *CPT* schema for each body cavity.

The panel developed FAST and E-FAST protocols to define ultrasonographic views and landmarks necessary to attain a complete positive or complete negative FAST or E-FAST result in children. In contrast to the adult protocols, the children’s protocols highlight the importance of the suprapubic view, which is the most sensitive for abdominal free fluid, especially in prepubescent children.^37^ In the round 1 survey, the panelists included more views and landmarks for defining a negative rather than a positive result. For example, only 1 view with intraperitoneal free fluid is necessary to define a positive abdominal FAST or E-FAST result. In contrast, all essential landmarks are important to define a complete negative FAST or E-FAST result. In addition, a negative result must be without free fluid in all required views. However, the panel noted that each view evaluates different anatomic regions; therefore, even an abdominal view with free fluid may still require cardiac and bilateral thoracic views. During the discussion, the panelists suggested that reporting FAST and E-FAST results should include any missing view or landmarks in the interpretation as well as patient factors (eg, stability, sex, and age) that could limit the examination. An example of a qualifying interpretation may state, “FAST result is negative but unable to visualize splenorenal recess in this unstable patient.” Future work should consider the development of a qualifying schema to standardize FAST and E-FAST reporting. Similarly, future investigation should focus on whether certain landmarks are more critical to view than others for a complete FAST.

Historically, FAST or E-FAST has been measured against improper reference standards. For example, the consensus panel found it inappropriate to use FAST or E-FAST to detect abdominal solid organ injury. Instead, FAST is used to detect intraperitoneal free fluid, which is assumed to be hemoperitoneum in the setting of trauma. This distinction is important because the FAST or E-FAST is not a replacement for CT scans but instead may be a tool within a diagnostic strategy for identifying the need for advanced imaging, resource utilization, or acute interventions.^38^ This distinction is important for future clinical and research purposes.

The panelists recognized that, most often in the suprapubic view, the FAST or E-FAST may show small volumes of physiological free fluid in some children.^32^ A few studies have suggested that clinicians who perform the FAST or E-FAST may accurately and reliably differentiate physiological from pathological free fluid.^39,40^ However, larger, more comprehensive studies would need to be completed before the panelists could agree that isolated, trace amounts of free fluid in the pelvis may be recognized as a qualified physiological finding by clinicians. Furthermore, the panelists emphasized the importance of clinical context and patient factors (eg, age and sex) when qualifying trace free fluid. Similarly, the panelists identified the dynamic opportunities of FAST and recognized that volume limits the detection of free fluid.^41,42^ In adult patients, 50 to 250 mL of free fluid must be present before it could be reliably detected on the FAST or E-FAST.^28^ To overcome the volume threshold barrier, the panelists suggested performing serial FAST studies to help improve overall accuracy by recognizing the dynamic changes in volume.^43^ Future studies should assess the accuracy and reliability of the FAST and E-FAST by defining trace free fluid and conducting serial studies.

### Limitations

This study has several limitations. First, the training and practice settings of this panel of POCUS experts may limit the generalizability of the findings. For the present modified Delphi technique, we included experts with substantial experience. Most panelists represented leaders in pediatric emergency POCUS within North America and were English speakers. However, these experts were from diverse geographic areas and represented highly respected institutions. Second, the focus of the consensus panel was to optimize the test characteristics of the FAST by defining an expert-level FAST protocol. The panel did not investigate clinical integration or address the psychomotor skills required for image acquisition.

## Conclusions

In this qualitative study, the expert panel achieved consensus on the definitions for complete FAST and E-FAST studies with high image quality and accurate image interpretation in children with injury. These definitions are similar to the adult protocol definitions. The consensus statements may be used for future education, quality assurance, and research. An agreement was reached on the potential use of serial FAST studies; however, the panelists were unclear on how to clinically interpret trace volumes of abdominal free fluid, suggesting a direction for future research.

## Notes

Supplement. **eTable 1.** Classification of Median Ratings **eTable 2.** Summary of Panel Responses of Appropriateness and Importance of Focused Assessment with Sonography for Trauma (FAST) in Children for Anatomic Views and Evaluations
