# Supplementary material for: Development of a Consensus-Based Definition of Focused Assessment With Sonography for Trauma in Children
Source: JAMA Netw Open. 2022 Mar 18;5(3):e222922. doi: 10.1001/jamanetworkopen.2022.2922 (PMC8933745; doi:10.1001/jamanetworkopen.2022.2922)
Supplement: Supplement. — eTable 1. Classification of Median Ratings eTable 2. Summary of Panel Responses of Appropriateness and Importance of Focused Assessment with Sonography for Trauma (FAST) in Children for Anatomic Views and Evaluations [file jamanetwopen-e222922-s001.pdf]

Supplementary Online Content

Kornblith AE, Addo N, Plasencia M, et al. Development of a consensus-based definition of Focused Assessment With Sonography for Trauma in children. *JAMA Netw Open*. 2022;5(3):e222922. doi:10.1001/jamanetworkopen.2022.2922

eTable 1. Classification of Median Ratings

eTable 2. Summary of Panel Responses of Appropriateness and Importance of Focused Assessment with Sonography for Trauma (FAST) in Children for Anatomic Views and Evaluations

This supplementary material has been provided by the authors to give readers additional information about their work.

© 2022 Kornblith AE et al. *JAMA Network Open.*

eTable 1. Classification of Median Ratings

Median Ratings	Appropriateness	Importance
**7 - 9** Without Disagreement	Appropriate	Important
**3.5 - 6.5** or any Disagreement	Uncertain	Uncertain
**1 - 3** Without Disagreement	Inappropriate	Not Important

Panel consensus was ascertained using the inter-percentile range adjusted for symmetry (IPRAS) method as described in the RAND/UCLA Appropriateness Method User's Manual (2001).

© 2022 Kornblith AE et al. *JAMA Network Open.*

eTable 2. Summary of Panel Responses of Appropriateness and Importance of Focused Assessment with Sonography for Trauma (FAST) in Children for Anatomic Views and Evaluations

**Anatomic Views**	FAST		Extended-FAST	
	Appropriateness Median (IQR)	Importance Median (IQR)	Appropriateness Median (IQR)	Importance Median (IQR)
Right Upper Abdominal Quadrant	9 (9 - 9)	9 (9 - 9)	9 (9 - 9)	9 (9 - 9)
Left Upper Abdominal Quadrant	9 (9 - 9)	9 (8.25 - 9)	9 (9 - 9)	9 (9 - 9)
Transverse Suprapubic	9 (9 - 9)	9 (8 - 9)	9 (9 - 9)	9 (8.25 - 9)
Sagittal Suprapubic	9 (9 - 9)	9 (9 - 9)	9 (9 - 9)	9 (9 - 9)
Lung/Pneumothorax	5 (2.25 - 6.75)	4 (1 - 5)	9 (9 - 9)	9 (9 - 9)
Subxiphoid Cardiac	9 (9 - 9)	9 (8 - 9)	9 (9 - 9)	9 (8 - 9)
Parasternal Long Cardiac	8 (5.25 - 9)	5.5 (3.25 - 7)	8 (7 - 9)	6.5 (5 - 8)
Parasternal Short Cardiac	5 (2.25 - 5.75)	3 (2 - 5)	5 (2.25 - 7)	3 (1.25 - 5)
Apical 4-Chamber Cardiac	3.5 (3 - 5)	2 (1 - 4)	3.5 (3 - 6)	2 (1 - 5)

IQR: interquartile range.

© 2022 Kornblith AE et al. *JAMA Network Open.*

eTable 2. Continued

**Evaluations**		
Importance<br>Median (IQR)	FAST	Extended-FAST
Intraperitoneal Free Fluid	9 (9 - 9)	9 (9 - 9)
Hemopericardium	9 (8 - 9)	9 (9 - 9)
Hemothoraces	9 (7 - 9)	9 (9 - 9)
Pneumothoraces	2 (1 - 4.75)	9 (9 - 9)
Cardiac Activity or Standstill	6 (4.25 -7.75)	6 (4.25 - 9)
Pneumopericardium	2 (1 - 4.75)	3 (2 - 6)
Bladder Injury	2 (1 - 4.75)	3 (1.25 - 6)

IQR: interquartile range.

© 2022 Kornblith AE et al. *JAMA Network Open.*
